# Susceptibility of widely diverse influenza a viruses to PB2 polymerase inhibitor pimodivir

**DOI:** 10.1016/j.antiviral.2021.105035

**Published:** 2021-02-10

**Authors:** Mira C. Patel, Anton Chesnokov, Joyce Jones, Vasiliy P. Mishin, Juan A. De La Cruz, Ha T. Nguyen, Natosha Zanders, David E. Wentworth, Todd C. Davis, Larisa V. Gubareva

**Affiliations:** a Influenza Division, National Center for Immunization and Respiratory Diseases, Centers for Disease Control and Prevention, Atlanta, GA, USA; b General Dynamics Information Technology, Atlanta, GA, USA

**Keywords:** Pimodivir, PB2 inhibitor, Drug susceptibility, Zoonotic influenza, H7N9, Pandemic potential

## Abstract

Pimodivir exerts an antiviral effect on the early stages of influenza A virus replication by inhibiting the cap-binding function of polymerase basic protein 2 (PB2). In this study, we used a combination of sequence analysis and phenotypic methods to evaluate pimodivir susceptibility of influenza A viruses collected from humans and other hosts. Screening PB2 sequences for substitutions previously associated with reduced pimodivir susceptibility revealed a very low frequency among seasonal viruses circulating in the U.S. during 2015–2020 (<0.03%; 3/11,934) and among non-seasonal viruses collected in various countries during the same period (0.2%; 18/8971). Pimodivir potently inhibited virus replication in two assays, a single-cycle HINT and a multi-cycle FRA, with IC_50_ values in a nanomolar range. Median IC_50_ values determined by HINT were similar for both subtypes of seasonal viruses, A(H1N1)pdm09 and A(H3N2), across three seasons. Human seasonal viruses with PB2 substitutions S324C, S324R, or N510K displayed a 27–317-fold reduced pimodivir susceptibility by HINT. In addition, pimodivir was effective at inhibiting replication of a diverse group of animal-origin viruses that have pandemic potential, including avian viruses of A(H5N6) and A(H7N9) subtypes. A rare PB2 substitution H357N was identified in an A(H4N2) subtype poultry virus that displayed >100-fold reduced pimodivir susceptibility. Our findings demonstrate a broad inhibitory activity of pimodivir and expand the existing knowledge of amino acid substitutions that can reduce susceptibility to this investigational antiviral.

## 1. Introduction

Influenza A viruses are respiratory pathogens of major economic and public health importance, because of their ability to cause high morbidity and mortality. Two subtypes of seasonal influenza A viruses, A(H1N1) and A(H3N2), have been causing recurrent epidemics of variable severity for several decades. Moreover, a vast natural reservoir for influenza A viruses of diverse subtypes exists in wild birds. Avian viruses are known to cause outbreaks with varying severity in humans and can also trigger a pandemic. Zoonotic infections with avian viruses of A (H5Nx), A(H7N9), A(H9N2) have been reported in recent years and pose a serious public health threat (Uyeki et al., 2017). The first influenza pandemic of the 21st century was caused by a swine-origin virus of A (H1N1) subtype, A(H1N1)pdm09, that suddenly emerged in North America in 2009 (Garten et al., 2009). In 2012, swine-origin A(H3N2) viruses caused a multi-state outbreak in the U.S. (Jhung et al., 2013). Swine-origin viruses recovered from humans are named “variant” (e.g., A(H3N2)v) to distinguish them from seasonal viruses. Furthermore, recently reported Eurasian avian-like swine A(H1N1) viruses with the A (H1N1)pdm09 M gene (i.e., genotype 4) also pose a threat to human health as they are antigenically distinct from previously circulating seasonal viruses and were reported to cause infections in humans in China at an increasing rate (Sun et al., 2020).

Vaccination is the principal tool to mitigate the burden of influenza. However, vaccine effectiveness can be reduced due to antigenic drift, or vaccines may not be available at the early stages of a pandemic. In such circumstances, antivirals can play an important role in controlling the impact of influenza, especially in populations that are at high-risk of developing complications.

Three classes of antiviral drugs – M2 blockers, neuraminidase (NA) inhibitors, and polymerase acidic (PA) inhibitor – are approved in numerous countries to control influenza A infections. However, M2 blockers are not recommended for use due to widespread resistance in contemporary seasonal influenza viruses. Although the M2 blocker amantadine has been used since 1970s, widespread resistance has occurred only after 2003 (Bright et al., 2005). The increased use of M2 blockers driven by the threat of bird flu and SARS-CoV in South East Asia during that time, was likely to contribute to the surge in M2 resistance in A(H3N2) viruses. Conversely, A(H1N1)pdm09 virus was already M2-resistant when it entered the human population, as it acquired the resistance-conferring M gene segment via reassortment with Eurasian swine influenza viruses (Garten et al., 2009). Since then, the oral NA inhibitor oseltamivir has been the most widely prescribed influenza antiviral. In 2007–2009, oseltamivir-resistant viruses of A(H1N1) subtype emerged and circulated globally until being displaced by the NA inhibitor-sensitive A(H1N1)pdm09 viruses (Hurt, 2014). The frequency of oseltamivir resistance in A(H1N1)pdm09 viruses has remained low, but transmission of such viruses in communities has been reported, indicating the need for continuous monitoring (McKimm-Breschkin, 2013; Takashita et al., 2020). The PA polymerase inhibitor, baloxavir entered the global market in 2018 and became a welcomed addition to the sparse toolbox of anti-influenza medications. However, baloxavir showed a low barrier to resistance emergence, with an especially high rate of resistance in young children (Hirotsu et al., 2019). In clinical studies, resistant influenza viruses emerged in ~2–20% of baloxavir-recipients (Hayden et al., 2018; Hirotsu et al., 2019). Baloxavir-resistant viruses have also been reported to efficiently transmit within close household settings (Imai et al., 2020; Takashita et al., 2019). In addition, reports on the detection of baloxavir-resistant viruses in children not treated with this antiviral, raises further public health concerns (Imai et al., 2020; Takashita et al., 2019; Yano et al., 2020). The ongoing concerns over resistance to available antivirals highlight the need for new therapeutics, preferably with different mechanisms of action.

Pimodivir (VX-787) is an orally administered inhibitor of the polymerase basic protein 2 (PB2). It prevents binding of PB2 to the 7-methyl GTP (m^7^ GTP) cap structures of host mRNA and inhibits early stages of viral transcription (Clark et al., 2014). Pimodivir occupies the central cap-binding domain of PB2 and interacts in a similar fashion to m^7^ GTP guanine base (Byrn et al., 2015). Analysis of X-ray crystallography structure revealed that multiple amino acid residues in cap- and mid-linker binding regions of PB2 extensively interact with pimodivir (Byrn et al., 2015; Ma et al., 2017). Due to structural differences in the PB2 cap-binding pocket of influenza A and B viruses, pimodivir is only active against influenza A viruses. Pimodivir was shown to be effective at inhibiting replication of seasonal influenza A and avian A(H5N1) viruses (Byrn et al., 2015; Clark et al., 2014). Pimodivir was advanced to late phase clinical development and was granted the fast track designation by the U.S. Food and Drug Administration (FDA) (Finberg et al., 2019; O’Neil et al., 2020; Trevejo et al., 2018).

A few studies have described limited information on amino acid substitutions associated with reduced susceptibility to pimodivir. A number of substitutions in PB2 protein have been reported to emerge readily under drug pressure *in vitro* (Byrn et al., 2015). In clinical studies, ~7–10% of pimodivir-recipients shed virus with substitutions in PB2 protein (Finberg et al., 2019; Trevejo et al., 2018). Previously reported amino acid substitutions occurred at nine residues positioned in mid, cap-binding, and RNA binding/linker regions of PB2 protein, with the majority residing in the cap-binding region (Byrn et al., 2015; Finberg et al., 2019; Trevejo et al., 2018) (Table 1). Notably, five amino acid substitutions were detected at a single residue, S324; some of them were seen in clinical trials. Multiple substitutions at two other residues, K376 and M431, were reported in pimodivir-treated patients. The emergence of viruses with substitutions at N510, which resides in the RNA-binding/linker region, were observed both in cell culture and clinical settings. The degree of pimodivir resistance varied depending on the substitution (Table 1). At this time, detailed phenotypic analysis describing impact of many of the amino acid substitutions detected in clinical trials was not reported.

In recent years, influenza antiviral susceptibility surveillance conducted by CDC has been primarily based on next generation sequencing (NGS) analysis, which is supplemented with phenotypic testing (Jester et al., 2018). In this study, we aimed to establish a methodology that is suitable for monitoring PB2 inhibitor resistance as part of virological surveillance conducted on seasonal and non-seasonal viruses.

## 2. Materials and methods

### 2.1. Viruses

Influenza viruses were submitted to the World Health Organization (WHO) Collaborating Center for Surveillance, Epidemiology and Control of Influenza at CDC by laboratories participating in the WHO Global Influenza Surveillance and Response System (GISRS). Viruses were propagated in Madin-Darby canine kidney (MDCK) cells (ATCC, Manassas, VA), MDCK-SIAT1 cells, or fertilized chicken eggs, depending on subtype and origin. All the procedures involving swine and low pathogenicity avian influenza viruses collected in the U.S. were conducted at biosafety level 2 enhanced containment, while other non-seasonal viruses were handled at biosafety level 3 enhanced containment.

### 2.2. Next generation sequencing analysis

Illumina MiSeq platform was utilized for codon-complete genome sequencing of influenza viruses and sequence analysis was performed as described (Shepard et al., 2016). Sequences have been made public through the Global Initiative on Sharing All Influenza Data (GISAID) and the NCBI influenza virus resource. Sequences of non-seasonal viruses were deposited to GISAID, after approval from the country of origin. Duplicative PB2 sequences for the same virus were removed before analysis.

### 2.3. Pimodivir susceptibility testing

Pimodivir was purchased from a commercial source (MedChemExpress), dissolved in DMSO as a 10 mM stock, aliquoted and stored at −80 °C. Viruses were tested by either focus reduction assay (FRA) or high-content imaging neutralization assay (HINT) as previously described (Gubareva et al., 2019; Marjuki et al., 2016). Both assays were carried out using MDCK-SIAT1 cells and serially diluted pimodivir (0.05–1000 nM). For FRA, cell monolayers prepared in a 24-well plate were inoculated with virus (30–60 foci per well) and incubated for 1 h at 37 °C. After removal of virus inoculum, 0.25% Avicel RC-591 overlay (FMC Biopolymer) in EMEM supplemented with pimodivir and TPCK-trypsin was added to the monolayers and plates were incubated at 37 °C and 5% CO_2_ for 18–24 h. For HINT assay, single-cell suspension was added to wells of a 96-well microplate containing a mixture of pimodivir and virus (~1000 infectious units) and microplates were incubated at 37 °C and 5% CO_2_ for 16–18 h; a single-cycle replication was achieved by omitting TPCK-trypsin from virus growth media.

Visualization of virus infected cells in both assays was done by immunostaining with mouse anti-viral nucleoprotein (NP) antibody (International Reagent Resource), followed by incubation with goat anti-mouse IgG antibody conjugated to Alexa Fluor-555 (ThermoFisher Scientific), and Hoechst 33,258 dye (AnaSpec Inc.) was used to stain cellular DNA. For FRA, the number of foci formed by virus-infected cells was counted under a fluorescence microscope; while for HINT, infected cell population was detected and quantified using either CellInsight CX5 (ThermoFisher Scientific) or Celigo (Nexcelom Bioscience) image cytometers. Pimodivir 50% inhibitory concentration (IC_50_) values were calculated by curve-fitting analysis.

Currently, there is no established criteria available for the definition of pimodivir resistance or reduced susceptibility. Therefore, we used the arbitrary criteria based on IC_50_ fold increase compared with the sequence matched control (wild-type) viruses from the same subtype. The arbitrary criteria define influenza virus inhibition as normal (≤3-fold increase) or reduced (>3-fold increase).

## 3. Results

### 3.1. PB2 sequence analysis of seasonal influenza A viruses, U.S., 2015–2020

In this study, NGS-generated PB2 sequences of 11,934 influenza A viruses were analyzed, which included 10,555 viruses collected by the U.S. virological surveillance during five recent seasons (October 1, 2015–April 17, 2020) and 1379 viruses collected for vaccine effectiveness studies during 2015–2017 seasons. Most (~61%) of the sequences belonged to the A(H3N2) subtype. Interrogation of PB2 sequences at nine residues of interest (Table 1) revealed an exceptionally low frequency of naturally occurring pimodivir resistance. Only three viruses (3/11,934, <0.03%) were identified to contain any of the PB2 substitutions (Table 2). Of these three, two A(H1N1)pdm09 viruses (A/Texas/70/2016 and A/Minnesota/11/2017) had S324C and N510K, respectively; while one A(H3N2) virus (A/Pennsylvania/242/2017) contained S324R. These substitutions were detected in both clinical specimens and respective virus isolates.

### 3.2. Phenotypic testing

To evaluate the effect of the identified PB2 substitutions on drug phenotype, the three flagged viruses were tested in cell culture with pimodivir. In addition, viruses with matching PB2 sequences, except the respective substitutions, were tested to calculate a fold increase in pimodivir IC_50_ value. In the multi-cycle replication-based FRA assay, S324C, N510K, and S324R conferred a 20-, 283-, and 688-fold reduction in pimodivir susceptibility, respectively (Table 2). Next, the same viruses were tested using a single-cycle replication-based assay, HINT, since this assay has been used to monitor susceptibility to PA inhibitor baloxavir (Gubareva et al., 2019). The HINT-generated pimodivir IC_50_ values were noticeably higher than those generated using FRA, however, the fold changes were similar (27-, 273-, and 317-fold reduction, respectively) (Table 2).

**Fig. 1.**
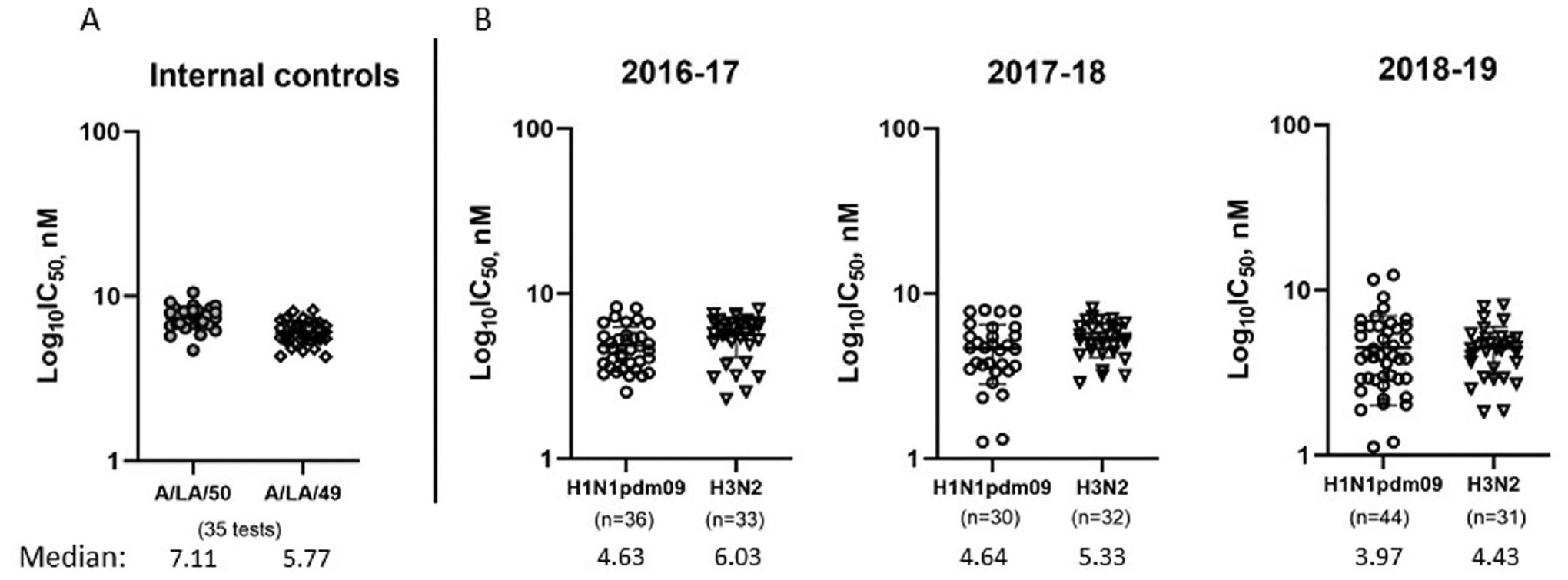
Pimodivir susceptibility of seasonal influenza A viruses circulating in the U.S. during 2016–2019 seasons. Viruses were tested by a single-cycle replication-based assay HINT in MDCK-SIAT1 cells and scatter plot of IC_50_ values are shown. (A) A(H3N2) viruses (A/Louisiana/50/2017-PA-I38 and A/Louisiana/49/2017-PA-I38M) were included in each test as internal controls. IC_50_ values of both viruses were plotted for 35 different tests, and their respective median values are shown. (B) Virus isolates of A (H1N1)pdm09 and A(H3N2) subtypes were tested to calculate median IC_50_ values across different seasons. Number of viruses tested and median values for each subtype in different seasons are shown. A single test result for each virus was used to compile IC_50_ results for each subtype. Few of the viruses giving IC_50_ in the outlier range, were re-tested to confirm the results.

As HINT offers an improved throughput, it was used to establish a pimodivir susceptibility baseline for viruses circulating in the U.S. during three recent seasons. As an internal quality control, a pair of A (H3N2) viruses was included in each test; their genomes were nearly identical, except substitution I38M in PA protein (Gubareva et al., 2019). Both control viruses showed consistent pimodivir IC_50_ values within and across multiple tests (Fig. 1A). However, the median pimodivir IC_50_ for the PA-I38M control virus was somewhat lower (5.77 vs 7.11 nM, p < 0.0001). As this mutation was shown to slow virus replication in MDCK-SIAT1 cells (Chesnokov et al., 2020), it might indirectly affect pimodivir IC_50_ values.

A total of 206 viruses representing A(H1N1)pdm09 (n = 110) and A (H3N2) (n = 96) subtypes collected during 2016–2019 were tested using HINT. The median subtype-specific IC_50_ values were similar, 4.46 nM vs 5.22 nM, respectively. It is worth noting that A(H1N1)pdm09 viruses displayed a somewhat broader IC_50_ range, 0.85–12.31 nM (~15-fold difference between the minimum and maximum), compared to A(H3N2) viruses (~5-fold range). Nevertheless, as shown in the scatter plots, subtype-specific IC_50_ values remained consistent between seasons (Fig. 1B).

### 3.3. Pimodivir susceptibility of non-seasonal influenza A viruses

We next interrogated 8971 PB2 sequences of non-seasonal viruses collected from human and non-human hosts between October 1, 2014–April 20, 2020 (accessed from GISAID on September 23, 2020). A total of 18 viruses carrying PB2 substitutions of concern were detected, indicating a low (18/8971; 0.20%) frequency of naturally occurring resistance to pimodivir. These substitutions were as follows: F325L (n = 1), S337P (n = 1), K376R (n = 3), T378S (n = 3), M431L (n = 3), and N510K (n = 7) (Supplementary Table 1). They were mainly found in avian viruses of A(H5Nx), A(H6N6), A(H7N3), and A(H9N2) subtypes. None of these viruses were available for phenotypic assessment.

Next, a panel of 23 ‘variant’ viruses of A(H1N1)v (n = 3), A(H1N2)v (n = 8), and A(H3N2)v (n = 12) subtypes was assembled for phenotypic testing using HINT. It comprised of viruses collected during outbreaks that took place in the U.S. during 2008–2018. All ‘variant’ viruses tested were resistant to M2 inhibitors due to the presence of V27T or S31N substitutions in M2 protein. The panel was complemented by classic swine influenza A(H1N1) viruses (n = 3). HINT pimodivir IC_50_ values ranged 1.12–27.77 nM (~25-fold range) with a median of 8.03 nM (Supplementary Table 2); A/swine/Tennessee/1/75 A(H1N1) displayed the lowest IC_50_, while A/Michigan/84/2016 A(H3N2)v had the highest. Notably, viruses from the same zoonotic outbreak showed similar IC_50_ values. For example, pimodivir IC_50_ range was much narrower, 5.40–10.10 nM, for five A(H1N2)v viruses collected during the 2018 zoonotic outbreak in California and Michigan. In addition, FRA was used to test a subset of viruses, including three A(H1N1)v collected in the U. S., Europe, and China (Supplementary Table 2). All tested viruses were sensitive to pimodivir (median IC_50_ 0.57 nM); similar to HINT results, the range by FRA was also broad (~35-fold range). The significant genetic heterogeneity of the viruses in the panel may be the underlining reason for the broad ranges of IC_50_ observed in both assays.

To assess pimodivir susceptibility of avian-origin viruses, we assembled a panel containing three subtypes of major epidemiological relevance: A(H7N9), A(H5N6), and A(H9N2) (Table 3). As highly pathogenic avian viruses do not require trypsin for replication in MDCK-SIAT1 cells, this panel was tested using FRA. Pimodivir potently inhibited replication of all the viruses tested at sub-nanomolar to low nanomolar concentrations; their genetic heterogeneity manifested in a wide range of IC_50_ values (~46-fold range) (Table 3).

Pimodivir effectively inhibited replication of a broad variety of swine and avian-origin influenza A viruses in cell culture.

### 3.4. Activity of pimodivir against viruses resistant to FDA-approved antivirals

Based on published data (Byrn et al., 2015) and the present study (Table 3, Supplementary Table 2), M2 blocker-resistant viruses are susceptible to pimodivir. We expanded this examination by assessing pimodivir activity against a panel of viruses displaying reduced susceptibility to one or more other approved antivirals, NA and PA inhibitors. Viruses of various subtypes carrying amino acid substitutions in NA and/or PA were selected from the CDC repository (n = 13) and tested using HINT. Their replication was inhibited by pimodivir at low nanomolar concentrations, 2.77–28.59 nM (Table 4), except one NA inhibitor-resistant virus, A/turkey/Minnesota/833/80 (H4N2) carrying NA-R292K mutation (ty/MN/80-NA-R292K) (Gubareva et al., 1996), which displayed highly elevated average pimodivir IC_50_, 644.17 nM. Notably, it’s NA inhibitor-sensitive counterpart (wild-type ty/MN/80-NA-R292) showed similarly high elevated average pimodivir IC_50_, 468.43 nM (data not shown).

### 3.5. Identification of a novel marker of pimodivir resistance

To elucidate the molecular basis for the observed pimodivir resistance of NA inhibitor-sensitive and -resistant ty/MN/80 viruses, their PB2 sequences were analyzed. None of the previously reported PB2 substitutions (Table 1) were found. However, two substitutions, H357N and L464M, residing in the cap-binding domain of the PB2, raised our interest. Histidine at position 357 was shown to participate in the stacking of pimodivir’s aromatic rings and making water-mediated interaction with a carboxylic acid of pimodivir (Clark et al., 2014; Byrn et al., 2015). Histidine is an aromatic amino acid, while asparagine is a polar amino acid with a positively charged side chain. Therefore, it is reasonable to assume that the substitution H357N may affect pimodivir susceptibility. PB2 sequences of 94,288 viruses (accessed from GISAID on September 23, 2020), including 63,923 (68%) viruses collected from humans, were analyzed for the presence of H357N. Notably, histidine at 357 was highly conserved (99.60%), and asparagine was present in only 22 viruses (20 of animal origin; 0.02%) (Supplementary Table 3). None of the H375N carrying viruses were available for testing. However, we were able to obtain and test two A(H1N1)pdm09 viruses, A/Manitoba/RV0444/2018 and A/Kuwait/3812/2017, that carry the second substitution of interest - L464M. Because their pimodivir IC_50_ values were low, average 3.48 nM, we concluded that H357N alone conferred the observed pimodivir resistance of ty/MN/80 virus.

## 4. Discussion

The PB2 inhibitor pimodivir showed significant anti-influenza efficacy in early clinical trials (Finberg et al., 2019; O’Neil et al., 2020; Trevejo et al., 2018). However, in the phase 3 study, pimodivir given in combination with the Standard-of-Care (influenza antivirals and/or supportive care only) did not show added benefits to hospitalized patients with influenza A infection (clinical trial identifier no. NCT03376321). The drug development program for pimodivir has been discontinued by the commercial developer (https://www.clinical-trialsarena.com/news/janssen-pimodivir-development/). Nonetheless, RNA-dependent RNA-polymerase remains an attractive target for antiviral development due to its critical roles in virus replication and the high degree of sequence conservation (Stevaert and Naesens, 2016). Therefore, the search for new polymerase inhibitors is likely to continue, including those targeting the cap-binding domain of PB2 (Chen et al., 2020; Tian et al., 2020). Our study reinforced the notion that naturally occurring resistance to pimodivir is rare in both seasonal and non-seasonal influenza A viruses. Insights into the molecular mechanisms of resistance to pimodivir may facilitate the development of PB2 inhibitors with improved efficacy.

The strength of this study is the use of a combination of sequence-based and phenotypic approaches, as well as testing genetically diverse influenza A viruses, including those with pandemic potential. IC_50_ values generated here using FRA were in agreement with a previous study (Byrn et al., 2015) also utilizing a multi-cycle based assay. Similar to neutralizing antibodies and baloxavir, pimodivir inhibits virus replication at an early stage. This trait allowed us to apply a single-cycle assay, HINT, to assess pimodivir susceptibility. HINT offers a stream-lined procedure by omitting the need for preparing/washing cell monolayer prior to infection and adding/removing semi-solid overlay; it has been used for monitoring antigenic drift and susceptibility to baloxavir (Jorquera et al., 2019; Gubareva et al., 2019). As was seen with baloxavir (Mishin et al., 2019), pimodivir IC_50_ values determined using HINT were higher compared to those generated using FRA. This is expected as it is more challenging to impede synthesis of viral NP protein under conditions of a single-cycle compared to multi-cycle infection. Despite these differences, fold changes in IC_50_ conferred by PB2 substitutions were similar between the two assays, making HINT a suitable method for detecting viruses displaying decreased pimodivir susceptibility.

Zoonotic infections with swine- and avian-origin viruses are concerning due to their pandemic potential. Swine-origin ‘variant’ viruses have caused multi-state outbreaks during the last two decades (Jhung et al., 2013) and are classified as a nationally notifiable infectious disease. The M gene of A(H1N1)pdm09 virus is now present in many swine viruses and this may increase the probability of them crossing the interspecies barrier (Lindstrom et al., 2012). The diversity of swine viruses has increased due to multiple reassortment events and this may explain the wide range of pimodivir IC_50_ observed in this study. Notably, pimodivir IC_50_ range for A(H3N2)v viruses is higher (2.10–27.77 nM) than other variant subtypes. However, not equal number of viruses were tested for each subtype. Moreover, for A(H1N2)v, out of eight viruses tested, five viruses were collected from same zoonotic outbreak. While in case of A(H3N2)v, we tested 12 viruses collected during different outbreaks in the U.S. from 2013 to 2017. Interestingly, two A(H3N2)v viruses (A/Ohio/27/2016 and A/Ohio/28/2016) collected from same outbreak, showed very similar pimodivir IC_50_ values (within ~2-fold range). As pimodivir impedes virus replication at an early stage, efficiency of virus internalization into cells may affect the IC_50_ values. Therefore, it is tempting to speculate that this range may reflect the difference in replicative fitness or internalization efficiency of these variant viruses in MDCK-SIAT1 cells, a cell line optimized for propagation of human viruses, which would require further experiments and would be focus of our future investigation. Consistent with this notion, seasonal A(H1N1)pdm09 viruses, which originated from swine, also displayed a broader range of IC_50_ values compared to A(H3N2) viruses. Moreover, it is noteworthy to mention that the PB2 gene segment of A (H1N1)pdm09 virus was derived from avian-origin viruses through reassortment (Garten et al., 2009).

While HINT has its advantages, FRA is better suited for testing highly pathogenic avian viruses and showed pimodivir to be highly potent. Interestingly, sequence analysis revealed that substitutions associated with pimodivir resistance were more likely to be found in viruses isolated from wild birds and poultry rather than seasonal viruses. In addition, characterization of viral replicative fitness due to PB2 amino acid substitutions associated with reduced drug susceptibility would be beneficial. However, reports on replicative fitness of pimodivir-resistant viruses remain sparse. In the phase 2a clinical trial of pimodivir, variant virus containing M431I was reported to display 12.5-fold reduced *in vitro* replication capacity compared to wild-type virus (Trevejo et al., 2018). One limitation of our study is that we did not analyze replicative fitness of pimodivir-resistant viruses in comparison to their wild-type controls, which would require further investigation. A potential role of H357N in pimodivir resistance was previously suggested, but no supporting phenotypic data was presented (Finberg et al., 2019). Our study showed that H357N, found in PB2 of ty/MN/80 viruses, indeed conferred a high level of pimodivir-resistance. Information on how H357N may affect viral fitness and pathogenicity is limited. Ty/MN/80 replicated efficiently in cell cultures (Gubareva et al., 1996). Zhu and colleagues demonstrated the role of H357N, which was originally found in a mouse adapted A(H1N1)pdm09 virus, on *in vitro* replicative fitness and pathogenicity in mice (Zhu et al., 2012). Engineered virus with H357N demonstrated replicative fitness comparable to a wild-type virus in human A549 cells, while its growth was elevated in porcine PK15 and murine LA-4 cells. Additionally, H357N enhanced polymerase activity in a mini-genome replication assay. Moreover, the engineered virus displayed markedly higher replication in mice lungs and mortality compared to wild-type virus. Even though amino acid polymorphism is rare at PB2 residues implicated in pimodivir resistance, existence of such viruses warrants the need for further investigations into their properties.

In summary, this study demonstrates the high potency of pimodivir as an inhibitor of influenza A virus replication in cell culture, including viruses with pandemic potential.

## Supplementary Material

Supp

## Figures and Tables

**Table 1:** PB2 amino acid substitutions previously associated with reduced susceptibility to pimodivir.

No.	PB2 amino acid position	Protein region	Amino acid substitution	Fold reduction in pimodivir susceptibility	Source	Reference
1	306	Mid	Q306H	186	*in vitro*	Byrn et al. (2015) ^a^
2	324	Cap-binding	S324I	157	*in vitro*	Byrn et al. (2015)
			S324N	127	*in vitro*	Byrn et al. (2015)
			S324R	63	*in vitro*	Byrn et al. (2015)
			S324C	not available	clinical	Trevejo et al. (2018) ^b^
			S324K/N/R	not specified^c^	clinical	Finberg et al. (2019) ^c^
3	325	Cap-binding	F325L	not specified	clinical	Finberg et al. (2019)
4	337	Cap-binding	S337P	not specified	clinical	Finberg et al. (2019)
5	376	Cap-binding	K376R	not available	clinical	Trevejo et al. (2018)
			K376N/R	not specified	clinical	Finberg et al. (2019)
6	378	Cap-binding	T378S	not specified	clinical	Finberg et al. (2019)
7	404	Cap-binding	F404Y	257	*in vitro*	Byrn et al. (2015)
8	431	Cap-binding	M431I	57	clinical	Trevejo et al. (2018)
			M431L/R/V	not available	clinical	Trevejo et al. (2018)
9	510	RNA binding/Linker	N510T	133	*in vitro*	Byrn et al. (2015)
			N510K	not specified	clinical	Finberg et al. (2019)

PB2: polymerase basic protein 2.

a In vitro selection of A/Puerto Rico/8/34 (H1N1) mutants in the presence of pimodivir. Pimodivir susceptibility of viruses was assessed using 3-day CPE assay.

b Experimental infection of volunteers with A/Wisconsin/67/2005 (H3N2); no information on phenotypic assay used is available.

c Adult patients naturally infected with either A(H1N1)pdm09 or A(H3N2) and treated with pimodivir. Subtype specific information for variant emergence is not available. Pimodivir susceptibility of viruses was assessed using ViroSpot assay in cell culture. A wide range of fold-reduced susceptibility (9.4 to >372.0) was provided without data for specific PB2 substitutions.

**Table 2:** Pimodivir susceptibility of seasonal influenza A viruses containing previously reported PB2 substitutions, U.S., 2015–2020.

Subtype	Virus name	PB2 amino acid substitution^a^		Codon^b^	FRA		HINT		
					IC_50_ nM Mean ± SD^c^	Fold increase^d^	IC_50_ nM Mean ± SD^c^	Fold increase^d^	Fold increase^e^
H1N1pdm09	A/Michigan/280/2017	S324	control	AGT	0.65 ± 0.13	1	4.36 ± 0.47	1	1
	A/Texas/70/2016	S324C	test	TGT	13.26 ± 1.13	20	116.06 ± 20.23	27	26
	A/South Dakota/13/2017	N510	control	AAC	0.73 ± 0.16	1	3.24 ± 0.74	1	1
	A/Minnesota/11/2017	N510K	test	AAA	206.44 ± 47.31	283	884.18 ± 80.54	273	198
H3N2	A/New Jersey/24/2017	S324	control	AGT	0.20 ± 0.05	1	4.22 ± 0.76	1	1
	A/Pennsylvania/242/2017^f^	S324R	test	AGA	136.39 ± 19.24	688	1338.24 ± 142.73	317	256

FRA: focus reduction assay; HINT: high-content imaging neutralization assay; IC_50_: 50% inhibitory concentration; PB2: polymerase basic protein 2; SD: standard deviation.

a PB2 sequences of control viruses are identical to test viruses, except for the indicated residue.

b Underlined base indicates the nucleotide change.

c Mean and SD of at least three independent tests.

d Fold change to IC_50_ of test virus compared with sequence-matched control virus.

e Fold change to IC_50_ of respective virus compared with the subtype-specific median values: 4.46 for A(H1N1)pdm09 and 5.22 for A(H3N2).

f A/Pennsylvania/242/2017 contained two additional substitutions, M2-K60R and HA2-E176D, compared to respective control virus.

**Table 3:** Assessment of pimodivir susceptibility of avian-origin influenza A viruses using FRA.

Subtype	Virus name	M2 blocker resistance marker in M2 protein	PB2 gene accession number	Pimodivir IC_50_ nM^a^
H5N6	A/Sichuan/26221/2014^b^	None	EPI533585	0.20; 0.20
	A/Yunnan/14563/2015^b^	S31N	EPI587618	0.39; 0.48
	A/chicken/Vietnam/NCVD-17A505/2017	None	EPI1815500	0.12; 0.11
	A/duck/Bangladesh/19D770/2017	None	EPI1330540	0.04; 0.05
H7N9 (wave 1)	A/Shanghai/2/2013	S31N	EPI439495	1.48; 1.50
H7N9 (wave 2)	A/Hong Kong/5942/2013	S31N	EPI490879	1.09; 0.77
	A/Hong Kong/2212982/2014	S31N	EPI502370	0.31; 0.41
	A/Hong Kong/734/2014	S31N	EPI498797	0.74; 1.07
H7N9 (wave 3)	A/Hong Kong/56/2015	S31N	EPI1489674	0.34; 0.26
	A/British Columbia/1/2015	S31N	EPI560395	0.29; 0.34
H7N9 (wave 4)	A/Hong Kong/793/2016	S31N	EPI1815507	1.83; 1.80
H7N9 (wave 5)	A/Hong Kong/125/2017	S31N	EPI977392	0.57; 0.65
	A/Hong Kong/214/2017	S31N	EPI884219	0.17; 0.23
H9N2	A/chicken/Vietnam/NCVD-LS14/2016	S31N	EPI1815492	0.31; 0.16

FRA: focus reduction assay; IC_50_: 50% inhibitory concentration; PB2: polymerase basic protein 2.

All procedures involving avian-origin viruses were conducted in biosafety level 3 enhanced containment. FRA was carried out using MDCK-SIAT1 cells.

a Replicate results.

b Highly pathogenic avian influenza virus.

**Table 4:** Antiviral activity of pimodivir against seasonal and non-seasonal viruses displaying reduced susceptibility to FDA-approved antivirals.

Subtype	Virus name	Amino acid substitution in NA^a^ or PA	Decreased susceptibility profile to antivirals^b^				PB2I^c^ Pimodivir IC50 nM
			NAI Oseltamivir	NAI Zanamivir	NAI Peramivir	PAI Baloxavir	
H1N1pdm09	A/Alabama/03/2020	NA-H274Y	X		X		2.78; 2.77
H3N2	A/Bethesda/956/2006	NA-R292K	X	X	X		4.92; 5.05
H3N2	A/Massachusetts/07/2013	NA-del245–248	X	X			5.23; 5.88
H3N2	A/Washington/33/2014	NA-E119V	X				4.02; 4.82
H3N2v	A/Ohio/88/2012	NA-S247P	X	X			24.12; 28.59
H4N2	A/turkey/Minnesota/833/80	NA-R292K	X	X	X		416.42; 871.91
H7N9	A/Shanghai/1/2013	NA-R292K	X				5.92; 5.46
H7N9	A/Taiwan/1/2013 clone 2	NA-E119V	X				5.64; 5.84
H7N9	A/Taiwan/1/2013 clone 5	NA-I222R	X	X	X		5.18; 3.48
H1N1pdm09	A/Illinois/37/2018	PA-I38L				X	7.94; 7.40
H3N2	A/Louisiana/49/2017	PA-I38M				X	6.13; 5.39
H3N2	A/Bangladesh/3007/2017	PA-I38T				X	5.77; 6.76
H1N1pdm09	A/Florida/20/2018	NA-H274Y & PA-E23G	X		X	X	5.09; 5.24

HINT: high-content imaging neutralization assay; IC_50_: 50% inhibitory concentration; NA: neuraminidase; PA: polymerase acidic protein.

NAI: NA inhibitor; PAI: PA inhibitor; PB2I: PB2 inhibitor.

a Amino acid number in NA is based on N2 amino acid numbering scheme.

b Decreased susceptibility of viurses is indicated by X. Decreased susceptibility to a NAI is defined as ≥ 10-fold increase in IC_50_ value determined using NA inhibition assay. Decreased susceptibility to PAI baloxavir is defined as ≥ 3-fold increase in IC_50_ value determined using HINT assay.

c IC_50_ values for pimodivir were determined using HINT. Replicate results are shown.

## References

[R1] Bright RA, Medina MJ, Xu X, Perez-Oronoz G, Wallis TR, Davis XM, Povinelli L, Cox NJ, Klimov AI, 2005. Incidence of adamantane resistance among influenza A (H3N2) viruses isolated worldwide from 1994 to 2005: a cause for concern. Lancet 366, 1175–1181. 16198766 10.1016/S0140-6736(05)67338-2

[R2] Byrn RA, Jones SM, Bennett HB, Bral C, Clark MP, Jacobs MD, Kwong AD, Ledeboer MW, Leeman JR, McNeil CF, Murcko MA, Nezami A, Perola E, Rijnbrand R, Saxena K, Tsai AW, Zhou Y, Charifson PS, 2015. Preclinical activity of VX-787, a first-in-class, orally bioavailable inhibitor of the influenza virus polymerase PB2 subunit. Antimicrob. Agents Chemother 59, 1569–1582. 25547360 10.1128/AAC.04623-14 PMC4325764

[R3] Chen F, Yang L, Zhai L, Huang Y, Chen F, Duan W, Yang J, 2020. Methyl brevifolincarboxylate, a novel influenza virus PB2 inhibitor from Canarium Album (Lour.) Raeusch. Chem. Biol. Drug Des 96, 1280–1291. 32519462 10.1111/cbdd.13740

[R4] Chesnokov A, Patel MC, Mishin VP, De La Cruz JA, Lollis L, Nguyen HT, Dugan V, Wentworth DE, Gubareva LV, 2020. Replicative fitness of seasonal influenza A viruses with decreased susceptibility to baloxavir. J. Infect. Dis 221, 367–371. 31541547 10.1093/infdis/jiz472 PMC8851376

[R5] Clark MP, Ledeboer MW, Davies I, Byrn RA, Jones SM, Perola E, Tsai A, Jacobs M, Nti-Addae K, Bandarage UK, Boyd MJ, Bethiel RS, Court JJ, Deng H, Duffy JP, Dorsch WA, Farmer LJ, Gao H, Gu W, Jackson K, Jacobs DH, Kennedy JM, Ledford B, Liang J, Maltais F, Murcko M, Wang T, Wannamaker MW, Bennett HB, Leeman JR, McNeil C, Taylor WP, Memmott C, Jiang M, Rijnbrand R, Bral C, Germann U, Nezami A, Zhang Y, Salituro FG, Bennani YL, Charifson PS, 2014. Discovery of a novel, first-in-class, orally bioavailable azaindole inhibitor (VX-787) of influenza PB2. J. Med. Chem 57, 6668–6678. 25019388 10.1021/jm5007275

[R6] Clinicaltrialsarenacom. Janssen to stop clinical development of pimodivir for influenza [Available from: https://www.clinicaltrialsarena.com/news/janssen-pimodivir-development/.

[R7] Finberg RW, Lanno R, Anderson D, Fleischhackl R, van Duijnhoven W, Kauffman RS, Kosoglou T, Vingerhoets J, Leopold L, 2019. Phase 2b study of pimodivir (JNJ-63623872) as monotherapy or in combination with oseltamivir for treatment of acute uncomplicated seasonal influenza A: TOPAZ trial. J. Infect. Dis 219, 1026–1034. 30428049 10.1093/infdis/jiy547

[R8] Garten RJ, Davis CT, Russell CA, Shu B, Lindstrom S, Balish A, Sessions WM, Xu X, Skepner E, Deyde V, Okomo-Adhiambo M, Gubareva L, Barnes J, Smith CB, Emery SL, Hillman MJ, Rivailler P, Smagala J, de Graaf M, Burke DF, Fouchier RA, Pappas C, Alpuche-Aranda CM, Lopez-Gatell H, Olivera H, Lopez I, Myers CA, Faix D, Blair PJ, Yu C, Keene KM, Dotson PD Jr., Boxrud D, Sambol AR, Abid SH, St George K, Bannerman T, Moore AL, Stringer DJ, Blevins P, Demmler-Harrison GJ, Ginsberg M, Kriner P, Waterman S, Smole S, Guevara HF, Belongia EA, Clark PA, Beatrice ST, Donis R, Katz J, Finelli L, Bridges CB, Shaw M, Jernigan DB, Uyeki TM, Smith DJ, Klimov AI, Cox NJ, 2009. Antigenic and genetic characteristics of swine-origin 2009 A(H1N1) influenza viruses circulating in humans. Science 325, 197–201. 19465683 10.1126/science.1176225 PMC3250984

[R9] Gubareva LV, Bethell R, Hart GJ, Murti KG, Penn CR, Webster RG, 1996. Characterization of mutants of influenza A virus selected with the neuraminidase inhibitor 4-guanidino-Neu5Ac2en. J. Virol 70, 1818–1827. 8627706 10.1128/jvi.70.3.1818-1827.1996 PMC190009

[R10] Gubareva LV, Mishin VP, Patel MC, Chesnokov A, Nguyen HT, De La Cruz J, Spencer S, Campbell AP, Sinner M, Reid H, Garten R, Katz JM, Fry AM, Barnes J, Wentworth DE, 2019. Assessing baloxavir susceptibility of influenza viruses circulating in the United States during the 2016/17 and 2017/18 seasons. Euro Surveill 24, 1800666. 10.2807/1560-7917.ES.2019.24.3.1800666 PMC6344838 30670144

[R11] Hayden FG, Sugaya N, Hirotsu N, Lee N, de Jong MD, Hurt AC, Ishida T, Sekino H, Yamada K, Portsmouth S, Kawaguchi K, Shishido T, Arai M, Tsuchiya K, Uehara T, Watanabe A, Baloxavir Marboxil Investigators G, 2018. Baloxavir marboxil for uncomplicated influenza in adults and adolescents. N. Engl. J. Med 379, 913–923. 30184455 10.1056/NEJMoa1716197

[R12] Hirotsu N, Sakaguchi H, Sato C, Ishibashi T, Baba K, Omoto S, Shishido T, Tsuchiya K, Hayden FG, Uehara T, Watanabe A, 2019. Baloxavir marboxil in Japanese pediatric patients with influenza: safety and clinical and virologic outcomes. Clin. Infect. Dis 71, 971–981. 10.1093/cid/ciz908 PMC7428393 31538644

[R13] Hurt AC, 2014. The epidemiology and spread of drug resistant human influenza viruses. Curr Opin Virol 8, 22–29. 24866471 10.1016/j.coviro.2014.04.009

[R14] Imai M, Yamashita M, Sakai-Tagawa Y, Iwatsuki-Horimoto K, Kiso M, Murakami J, Yasuhara A, Takada K, Ito M, Nakajima N, Takahashi K, Lopes TJS, Dutta J, Khan Z, Kriti D, van Bakel H, Tokita A, Hagiwara H, Izumida N, Kuroki H, Nishino T, Wada N, Koga M, Adachi E, Jubishi D, Hasegawa H, Kawaoka Y, 2020. Influenza A variants with reduced susceptibility to baloxavir isolated from Japanese patients are fit and transmit through respiratory droplets. Nat Microbiol 5, 27–33. 31768027 10.1038/s41564-019-0609-0 PMC13014278

[R15] Jester B, Schwerzmann J, Mustaquim D, Aden T, Brammer L, Humes R, Shult P, Shahangian S, Gubareva L, Xu X, Miller J, Jernigan D, 2018. Mapping of the US domestic influenza virologic surveillance landscape. Emerg. Infect. Dis 24, 1300–1306. 10.3201/eid2407.180028 PMC6038762 29715078

[R16] Jhung MA, Epperson S, Biggerstaff M, Allen D, Balish A, Barnes N, Beaudoin A, Berman L, Bidol S, Blanton L, Blythe D, Brammer L, D’Mello T, Danila R, Davis W, de Fijter S, Diorio M, Durand LO, Emery S, Fowler B, Garten R, Grant Y, Greenbaum A, Gubareva L, Havers F, Haupt T, House J, Ibrahim S, Jiang V, Jain S, Jernigan D, Kazmierczak J, Klimov A, Lindstrom S, Longenberger A, Lucas P, Lynfield R, McMorrow M, Moll M, Morin C, Ostroff S, Page SL, Park SY, Peters S, Quinn C, Reed C, Richards S, Scheftel J, Simwale O, Shu B, Soyemi K, Stauffer J, Steffens C, Su S, Torso L, Uyeki TM, Vetter S, Villanueva J, Wong KK, Shaw M, Bresee JS, Cox N, Finelli L, 2013. Outbreak of variant influenza A(H3N2) virus in the United States. Clin. Infect. Dis 57, 1703–1712. 24065322 10.1093/cid/cit649 PMC5733625

[R17] Jorquera PA, Mishin VP, Chesnokov A, Nguyen HT, Mann B, Garten R, Barnes J, Hodges E, De La Cruz J, Xu X, Katz J, Wentworth DE, Gubareva LV, 2019. Insights into the antigenic advancement of influenza A(H3N2) viruses, 2011–2018. Sci. Rep 9, 2676. 30804469 10.1038/s41598-019-39276-1 PMC6389938

[R18] Lindstrom S, Garten R, Balish A, Shu B, Emery S, Berman L, Barnes N, Sleeman K, Gubareva L, Villanueva J, Klimov A, 2012. Human infections with novel reassortant influenza A(H3N2)v viruses, United States, 2011. Emerg. Infect. Dis 18, 834–837. 22516540 10.3201/eid1805.111922 PMC3358066

[R19] Ma X, Xie L, Wartchow C, Warne R, Xu Y, Rivkin A, Tully D, Shia S, Uehara K, Baldwin DM, Muiru G, Zhong W, Zaror I, Bussiere DE, Leonard VHJ, 2017. Structural basis for therapeutic inhibition of influenza A polymerase PB2 subunit. Sci. Rep 7, 9385. 28839261 10.1038/s41598-017-09538-x PMC5571044

[R20] Marjuki H, Mishin VP, Chai N, Tan MW, Newton EM, Tegeris J, Erlandson K, Willis M, Jones J, Davis T, Stevens J, Gubareva LV, 2016. Human monoclonal antibody 81.39a effectively neutralizes emerging influenza A viruses of group 1 and 2 hemagglutinins. J. Virol 90, 10446–10458. 27630240 10.1128/JVI.01284-16 PMC5110155

[R21] McKimm-Breschkin JL, 2013. Influenza neuraminidase inhibitors: antiviral action and mechanisms of resistance. Influenza Other Respir Viruses 7 (Suppl. 1), 25–36. 23279894 10.1111/irv.12047 PMC4942987

[R22] Mishin VP, Patel MC, Chesnokov A, De La Cruz J, Nguyen HT, Lollis L, Hodges E, Jang Y, Barnes J, Uyeki T, Davis CT, Wentworth DE, Gubareva LV, 2019. Susceptibility of influenza A, B, C, and D viruses to baloxavir. Emerg. Infect. Dis 25, 1969–1972. 31287050 10.3201/eid2510.190607 PMC6759234

[R23] O’Neil B, Ison MG, Hallouin-Bernard MC, Nilsson AC, Torres A, Wilburn JM, van Duijnhoven W, Van Dromme I, Anderson D, Deleu S, Kosoglou T, Vingerhoets J, Rossenu S, Leopold L, 2020. A phase 2 study of pimodivir (JNJ-63623872) in combination with oseltamivir in elderly and NonElderly adults hospitalized with influenza A infection: OPAL study. J. Infect. Dis10.1093/infdis/jiaa376 PMC9373154 32604406

[R24] Shepard SS, Meno S, Bahl J, Wilson MM, Barnes J, Neuhaus E, 2016. Viral deep sequencing needs an adaptive approach: IRMA, the iterative refinement meta-assembler. BMC Genom 17, 708. 10.1186/s12864-016-3030-6 PMC5011931 27595578

[R25] Stevaert A, Naesens L, 2016. The influenza virus polymerase complex: an update on its structure, functions, and significance for antiviral drug design. Med. Res. Rev 36, 1127–1173. 27569399 10.1002/med.21401 PMC5108440

[R26] Sun H, Xiao Y, Liu J, Wang D, Li F, Wang C, Li C, Zhu J, Song J, Sun H, Jiang Z, Liu L, Zhang X, Wei K, Hou D, Pu J, Sun Y, Tong Q, Bi Y, Chang KC, Liu S, Gao GF, Liu J, 2020. Prevalent Eurasian avian-like H1N1 swine influenza virus with 2009 pandemic viral genes facilitating human infection. Proc. Natl. Acad. Sci. U. S. A 117, 17204–17210. 32601207 10.1073/pnas.1921186117 PMC7382246

[R27] Takashita E, Ichikawa M, Morita H, Ogawa R, Fujisaki S, Shirakura M, Miura H, Nakamura K, Kishida N, Kuwahara T, Sugawara H, Sato A, Akimoto M, Mitamura K, Abe T, Yamazaki M, Watanabe S, Hasegawa H, Odagiri T, 2019. Human-to-Human transmission of influenza A(H3N2) virus with reduced susceptibility to baloxavir, Japan, february 2019. Emerg. Infect. Dis 25, 2108–2111. 31436527 10.3201/eid2511.190757 PMC6810216

[R28] Takashita E, Daniels RS, Fujisaki S, Gregory V, Gubareva LV, Huang W, Hurt AC, Lackenby A, Nguyen HT, Pereyaslov D, Roe M, Samaan M, Subbarao K, Tse H, Wang D, Yen HL, Zhang W, Meijer A, 2020. Global update on the susceptibilities of human influenza viruses to neuraminidase inhibitors and the cap-dependent endonuclease inhibitor baloxavir, 2017–2018. Antivir. Res 175, 104718. 32004620 10.1016/j.antiviral.2020.104718

[R29] Tian Y, Sang H, Liu M, Chen F, Huang Y, Li L, Liu S, Yang J, 2020. Dihydromyricetin is a new inhibitor of influenza polymerase PB2 subunit and influenza-induced inflammation. Microb. Infect 22, 254–262. 10.1016/j.micinf.2020.05.021 32554102

[R30] Trevejo JM, Asmal M, Vingerhoets J, Polo R, Robertson S, Jiang Y, Kieffer TL, Leopold L, 2018. Pimodivir treatment in adult volunteers experimentally inoculated with live influenza virus: a Phase IIa, randomized, double-blind, placebo-controlled study. Antivir. Ther 23, 335–344. 29244026 10.3851/IMP3212

[R31] Uyeki TM, Katz JM, Jernigan DB, 2017. Novel influenza A viruses and pandemic threats. Lancet 389, 2172–2174. 28589883 10.1016/S0140-6736(17)31274-6 PMC6637738

[R32] Yano T, Ochiai H, Akachi S, Matsumura Y, 2020. Polymerase acidic subunit I38T mutant influenza A (H3N2) virus isolated from a pediatric patient without prior baloxavir marboxil treatment in Mie Prefecture (November 2018). Jpn. J. Infect. Dis 73, 383–385. 32475868 10.7883/yoken.JJID.2019.210

[R33] Zhu W, Zhu Y, Qin K, Yu Z, Gao R, Yu H, Zhou J, Shu Y, 2012. Mutations in polymerase genes enhanced the virulence of 2009 pandemic H1N1 influenza virus in mice. PloS One 7, e33383. 22438920 10.1371/journal.pone.0033383 PMC3305307

