# Supplementary material for: Susceptibility of widely diverse influenza a viruses to PB2 polymerase inhibitor pimodivir
Source: Antiviral Res. Author manuscript; Available in PMC 2022 Apr 4. (PMC8978222; doi:10.1016/j.antiviral.2021.105035)
Supplement: Supp [file NIHMS1791117-supplement-Supp.docx]

Supplementary Table 1: PB2 amino acid substitutions previously associated with decreased pimodivir susceptibility: non-seasonal influenza A viruses, 2014-2020

| PB2 amino acid substitution | Subtype | Virus name | Species | PB2 gene accession number |
| --- | --- | --- | --- | --- |
| F325L | H5N1 | A/whooper swan/Henan/SMX9/2015 | avian | EPI559888 |
| S337P | H5N6 | A/goose/Hunan/HN22/2015 | avian | EPI958693 |
| K376R | H1N1 | A/swine/France/24-170162/2017 | swine | EPI1773475 |
|  | H7N3 | A/chicken/Guanajuato/CPA-02921-16-CENASA-95294/2016 | avian | EPI778523 |
|  | H7N3 | A/chicken/Jalisco/CPA-04173-16-CENASA-95294/2016 | avian | EPI778530 |
| T378S | H5N2 | A/duck/Hubei/SZY250/2016 | avian | EPI823937 |
|  | H5N6 | A/chicken/Guangdong/04.15 SZBAXQ019/2015 | avian | EPI661916 |
|  | H5N6 | A/oriental magpie robin/HK/6154/2015 | avian | EPI1060772 |
| M431L | H5N2 | A/American wigeon/California/LS257/2014 | avian | EPI812867 |
|  | H6N6 | A/chicken/Zhejiang/514132/2015 | avian | EPI753488 |
|  | H6N6 | A/chicken/Zhejiang/514158/2015 | avian | EPI753489 |
| N510K | H5N6 | A/duck/Yunnan/YN-2/2016 | avian | EPI1091673 |
|  | H9N2 | A/chicken/Viet Nam/QN-2576/2015 | avian | EPI1576759 |
|  | H9N2 | A/chicken/Viet Nam/QN-2577/2015 | avian | EPI1576585 |
|  | H9N2 | A/chicken/Viet Nam/QN-2579/2015 | avian | EPI1576525 |
|  | H10N5 | A/environment/New Jersey/UGAI16-2319/2016 | unknown | EPI1065961 |
|  | H10N5 | A/environment/New Jersey/UGAI16-2321/2016 | unknown | EPI1065969 |
|  | H10N5 | A/environment/New Jersey/UGAI16-2322/2016 | unknown | EPI1065977 |

Supplementary Table 2: Swine-origin influenza A viruses tested for pimodivir susceptibility using HINT and FRA

| Subtype | Virus name | M2 blocker resistance marker in M2 protein | PB2 gene accession number | Pimodivir IC_50_ nM^a^ |
| --- | --- | --- | --- | --- |
| **Tested by HINT^b^** | | | | |
| H1N1 | A/swine/Iowa/15/30 | none | EPI130317 | 7.42; 9.70 |
|  | A/swine/1976/31 | none | EPI242597 | 4.80; 4.30 |
|  | A/swine/Tennessee/1/75 | none | EPI62689 | 1.12; 1.24 |
| H1N1v | A/South Dakota/03/2008 | V27T | EPI291880 | 19.44; 17.87 |
|  | A/Texas/14/2008 | V27T | EPI291888 | 10.74; 7.95 |
|  | A/Minnesota/33/2014 | S31N | EPI558604 | 3.10; 2.59 |
| H1N2v | A/Ohio/24/2017 | S31N | EPI1056722 | 2.72; 2.57 |
|  | A/Ohio/35/2017 | S31N | EPI1056730 | 12.38 |
|  | A/Ohio/25/2018 | S31N | EPI1311350 | 3.00 |
|  | A/California/62/2018 | S31N | EPI1311358 | 9.60 |
|  | A/California/63/2018 | S31N | EPI1311366 | 10.10 |
|  | A/Michigan/382/2018 | S31N | EPI1271031 | 6.60 |
|  | A/Michigan/383/2018 | S31N | EPI1271039 | 6.20; 5.40 |
|  | A/Michigan/384/2018 | S31N | EPI1271063 | 7.50 |
| H3N2v | A/Iowa/04/2013 | S31N | EPI516841 | 8.61; 9.32 |
|  | A/Ohio/4319/2014 | S31N | EPI541957 | 21.34; 17.61 |
|  | A/Wisconsin/24/2014 | S31N | EPI557539 | 10.22; 11.67 |
|  | A/Ohio/02/2014 | S31N | EPI539156 | 6.81 |
|  | A/Michigan/39/2015 | S31N | EPI642510 | 13.06 |
|  | A/Michigan/83/2016 | S31N | EPI824772 | 24.36; 17.35 |
|  | A/Michigan/84/2016 | S31N | EPI838248 | 20.95; 27.77 |
|  | A/Ohio/28/2016 | S31N | EPI824754 | 3.98 |
|  | A/Ohio/27/2016 | S31N | EPI824746 | 9.31; 5.26 |
|  | A/North Dakota/19/2017 | S31N | EPI1311462 | 19.94; 14.99 |
|  | A/Ohio/15/2017 | S31N | EPI1056666 | 7.30; 7.64 |
|  | A/Ohio/29/2017 | S31N | EPI1311406 | 2.61; 2.10 |
| **Tested by FRA^c^** | | | | |
| H1N1 | A/swine/Tennessee/1/75 | none | EPI62689 | 0.07; 0.07 |
| H1N1v | A/Minnesota/33/2014 | S31N | EPI558604 | 0.60; 0.62 |
|  | A/Hunan/42443/2015 | S31N | EPI691392 | 0.35; 0.46 |
|  | A/Netherlands/3315/2016 | S31N | EPI888681 | 0.25, 0.26 |
| H3N2v | A/Ohio/4319/2014 | S31N | EPI541957 | 1.95; 2.46 |
|  | A/Michigan/83/2016 | S31N | EPI824772 | 0.80; 0.68 |
|  | A/Michigan/84/2016 | S31N | EPI838248 | 1.44; 1.52 |
|  | A/Ohio/29/2017 | S31N | EPI1311406 | 0.50; 0.55 |

FRA: focus reduction assay; HINT: high-content imaging neutralization assay; PB2: polymerase basic protein 2

^a^For most viruses replicate IC_50_ values are shown. Single IC_50_ value is shown for viruses which were tested once.

^b^Median IC_50_ value for swine-origin viruses tested by HINT is 8.03 nM.

^c^Median IC_50_ value for swine-origin viruses tested by FRA is 0.57 nM.

Supplementary Table 3: Influenza A viruses carrying PB2-H357N (GISAID database, 23 September 2020)

| Virus name | Subtype | Species | PB2 gene accession number |
| --- | --- | --- | --- |
| A/Taiwan/107/2006 | H1N1 | mammalian | EPI443882 |
| A/mallard/Maryland/322/2002 | H1N1 | avian | EPI285761 |
| A/mallard/Maryland/334/2002 | H1N1 | avian | EPI285762 |
| A/Washington/214/2017 | H3N2 | mammalian | EPI1109899 |
| A/green-winged teal/Interior Alaska/6MP0736/2006 | H3N8 | avian | EPI298505 |
| A/mallard/Interior Alaska/6MP0272/2006 | H3N8 | avian | EPI298641 |
| A/mallard/Interior Alaska/6MP0972R1/2006 | H3N8 | avian | EPI298737 |
| A/turkey/Minnesota/833/1980 | H4N2 | avian | EPI1565960 |
| A/turkey/Minnesota/833/1980 | H4N2 | avian | EPI1565968 |
| A/mallard/Vietnam/3/2003 | H5N1 | avian | EPI103457 |
| A/chicken/Vietnam/4/2003 | H5N1 | avian | EPI103459 |
| A/chicken/Vietnam/5/2003 | H5N1 | avian | EPI103461 |
| A/chicken/Vietnam/8/2003 | H5N1 | avian | EPI103463 |
| A/chicken/TongHai/302/2014 | H5N1 | avian | EPI585948 |
| A/domestic goose/Poland/69/2017 | H5N8 | avian | EPI1184631 |
| A/anser fabalis/China/Anhui/L63/2014 | H6N2 | avian | EPI895303 |
| A/mallard/Southcentral Alaska/15ML00267/2015 | H7N3 | avian | EPI762407 |
| A/mallard/Kentucky/AH0051797/2016 | H7N3 | avian | EPI952736 |
| A/green-winged teal/Alaska/AK18-WB1-015A/2018 | H8N4 | avian | EPI1772755 |
| A/mink/China/01/2014 | H9N2 | mammalian | EPI1086308 |
| A/ruddy turnstone/New Jersey/238/2004 | H10N7 | avian | EPI158734 |
| A/shorebird/Delaware Bay/322/2009 | H10N7 | avian | EPI436017 |
